# Heatwaves in Peninsular Malaysia: a spatiotemporal analysis

**DOI:** 10.1038/s41598-024-53960-x

**Published:** 2024-02-21

**Authors:** Mohd Khairul Idlan Muhammad, Mohammed Magdy Hamed, Sobri Harun, Zulfaqar Sa’adi, Saad Sh. Sammen, Nadhir Al-Ansari, Shamsuddin Shahid, Miklas Scholz

**Affiliations:** 1 Department of Water and Environmental Engineering, Faculty of Civil Engineering, Universiti Teknologi Malaysia (UTM), 81310 Skudai, Johor, Malaysia; 2 Construction and Building Engineering Department, College of Engineering and Technology, Arab Academy for Science, Technology and Maritime Transport (AASTMT), B 2401 Smart Village, Giza, 12577 Egypt; 3 Centre for Environmental Sustainability and Water Security (IPASA), Research Institute for Sustainable Environment (RISE), Universiti Teknologi Malaysia, UTM, 81310 Skudai, Johor, Malaysia; 4 Department of Civil Engineering, College of Engineering, Diyala University, Baqubah, 32001 Iraq; 5 Civil, Environmental and Natural Resources Engineering, Lulea University of Technology, 97187 Lulea, Sweden; 6 Environmental and Atmospheric Sciences Research Group, Scientific Research Center, Al-Ayen University, Thi-Qar, Nasiriyah, 64001 Iraq; 7 Innovation Management Department, Atene KOM, Invalidenstraße 91, 10115 Berlin, Germany; 8 Department of Civil Engineering Science, Faculty of Engineering and the Built Environment, School of Civil Engineering, and the Built Environment, University of Johannesburg, Kingsway Campus, Aukland Park, PO Box 524, Johannesburg, 2006 South Africa; 9 School of Science, Engineering and Environment, The University of Salford, Newton Building, Greater Manchester, M5 4WT UK; 10 Specialist Company According to Water Law, Kunststoff-Technik Adams, Schulstraße 7, 26931 Elsfleth, Germany; 11 Nexus By Sweden, Skepparbacken 5, 722 11 Västerås, Sweden; 12 Department of Town Planning, Engineering Networks and Systems, South Ural State University (National Research University), 76, Lenin Prospekt, Chelyabinsk, Russia 454080

**Keywords:** Hot extremes, Reanalysis data, LandScan population, Tropical region, Peninsular Malaysia, Climate sciences, Atmospheric science, Climate change, Environmental impact

## Abstract

One of the direct and unavoidable consequences of global warming-induced rising temperatures is the more recurrent and severe heatwaves. In recent years, even countries like Malaysia seldom had some mild to severe heatwaves. As the Earth's average temperature continues to rise, heatwaves in Malaysia will undoubtedly worsen in the future. It is crucial to characterize and monitor heat events across time to effectively prepare for and implement preventative actions to lessen heatwave's social and economic effects. This study proposes heatwave-related indices that take into account both daily maximum (Tmax) and daily lowest (Tmin) temperatures to evaluate shifts in heatwave features in Peninsular Malaysia (PM). Daily ERA5 temperature dataset with a geographical resolution of 0.25° for the period 1950–2022 was used to analyze the changes in the frequency and severity of heat waves across PM, while the LandScan gridded population data from 2000 to 2020 was used to calculate the affected population to the heatwaves. This study also utilized Sen's slope for trend analysis of heatwave characteristics, which separates multi-decadal oscillatory fluctuations from secular trends. The findings demonstrated that the geographical pattern of heatwaves in PM could be reconstructed if daily Tmax is more than the 95th percentile for 3 or more days. The data indicated that the southwest was more prone to severe heatwaves. The PM experienced more heatwaves after 2000 than before. Overall, the heatwave-affected area in PM has increased by 8.98 km^2^/decade and its duration by 1.54 days/decade. The highest population affected was located in the central south region of PM. These findings provide valuable insights into the heatwaves pattern and impact.

## Introduction

The shift in temperature mean and variability due to climate change caused a rapid rise in all temperature-related extremes^1,2^. Numerous studies have indicated exacerbated heatwave events due to climate change, particularly in duration and intensity^3–6^. The increased frequency and severity of heatwaves are most imminent, which would inadvertently affect the ecosystem, infrastructure, human health, and social life^7–10^. It has been reported that the present global heatwave frequency is five times more than the pre-industrial period^11^. Higher populations vulnerable to heatwaves increase the risk of disease and death^12,13^. This has been noticeable globally in the last two decades^14–16^.

The increase in temperature and heatwaves are not homogeneous^9,17^. Instead, regional solid heterogeneity has been noticed. The most considerable rise in heatwaves is reported in the Middle East and parts of Africa and South America^2,18–21^. The heatwaves also increased rapidly in some Asian countries, particularly South and Southeast Asia countries^3,17,22^. Perkins-Kirkpatrick and Lewis^23^ reported an increase in heatwave days in Southeast Asia by 4.2 days/decade in contrast to the global mean of 2.26 days/decade. Xian et al.^24^ reported a strong increasing trend in different characteristics of Southeast Asia's heatwaves from 1979 to 2018, with the highest increase in the Malay and Indochina Peninsulas.

In Peninsular Malaysia (PM), the temperature-related extreme events are relatively low due to the low annual and seasonal variability of temperature (± 2 °C)^25^. However, PM has experienced a temperature rise of 0.18 °C/decade since 1951^22,26^. Climate models also projected an acceleration of rising temperatures in the future. MMD^27^ projected a future rise in PM temperature by 1.1 to 3.6 °C during 2070–2099. Paterson, et al.^28^ projected that rising temperatures would increase heat stress in PM and nearby regions. Temperature variability in PM also increased in the line of global temperature, causing a rise in temperature extremes and heatwaves. Consequently, PM experienced a few heatwaves in recent years that it had never experienced earlier. The maximum temperature (Tmax) in some northern states increased above 37 °C and persisted for 3 days in April 2016, which compelled the local authorities to close the school and ban outdoor activities. The daily Tmax in some Southeast Asian countries also increased up to 45 °C during that period, which caused a devastating heatwave over the region^3,20^. In March 2019, most PM experienced temperatures between 35 and 37 °C for three consecutive days^29^. In some areas in the north, the temperature exceeded 40 °C, severely disturbing daily activities. A similar situation happened in the north of PM in March 2020. The frequent occurrence of heatwaves in recent years provides evidence of climate change in PM. Therefore, it is vital to evaluate the ongoing changes in heatwave characteristics and the temperature characteristics responsible for its occurrence. Such information can help to anticipate future changes in the heatwaves and decide the necessary adaptation measures to mitigate its effects on society. The literature related to heatwaves in Malaysia is very few. Despite no common definition, heatwave is generally denoted as an abnormally higher temperature for a few days^22^. Malaysia has already established a specific definition for different forms of heatwaves. Table 1 shows the different levels of heatwaves based on temperature thresholds and suggested actions to combat them.

**Table 1:** Heatwave levels of peninsular Malaysia^30^.

Level	Threshold	Action
1	Tmax between 35.0 and 37.0 °C for 3 days in a row	Alert
2	Tmax between 37.0 and 40.0 °C for 3 days in a row	Announcement of heatwave to take actions, such as the closure of schools and so on
3	Tmax exceeds 40.0 °C for 3 days in a row	Announcement of a state of emergency

Heatwave impacts vary from place to place, and thus, the definition. Many definitions have been made for heatwave analysis in different regions of the globe^31^. Heatwaves are most widely defined according to area, geography, and time, where it is an event when Tmax exceeds a certain threshold for a few consecutive days^32–34^. However, there is a dispute about the threshold and the number of consecutive days. Some heatwave definitions are based on an absolute threshold, such as 37 °C is considered the threshold for defining heatwave in Malaysia^35^. Some of the definitions are based on percentile, such as more than the 95th percentile temperature of a predefined reference period^35^. Generally, the threshold is decided according to human health consequences^8,33,36–40^. On the other hand, a set of weather elements is linked to the human heat stress levels to define heatwave at a regional level^7,41–43^.

The main drawback of defining heatwaves according to human health consequences is that it is not usable in assessing heatwave alteration over time or comparing heatwave severity between two geographical regions. This is because they are defined according to a relationship in a reference period, thus making them unsuitable for generating heatwave time series for change analysis^31^. Therefore, this study attempted to evaluate the temporal and geographical distribution of different heatwave characteristics in PM based on different temperature thresholds. This is the first study of heatwaves assessment in PM regarding the affected area, duration, severity and heat wave index. This study would assist in understanding the changes in heatwave characteristics and gaining insight into heatwave definitions for PM.

## Study area

**Figure 1:**
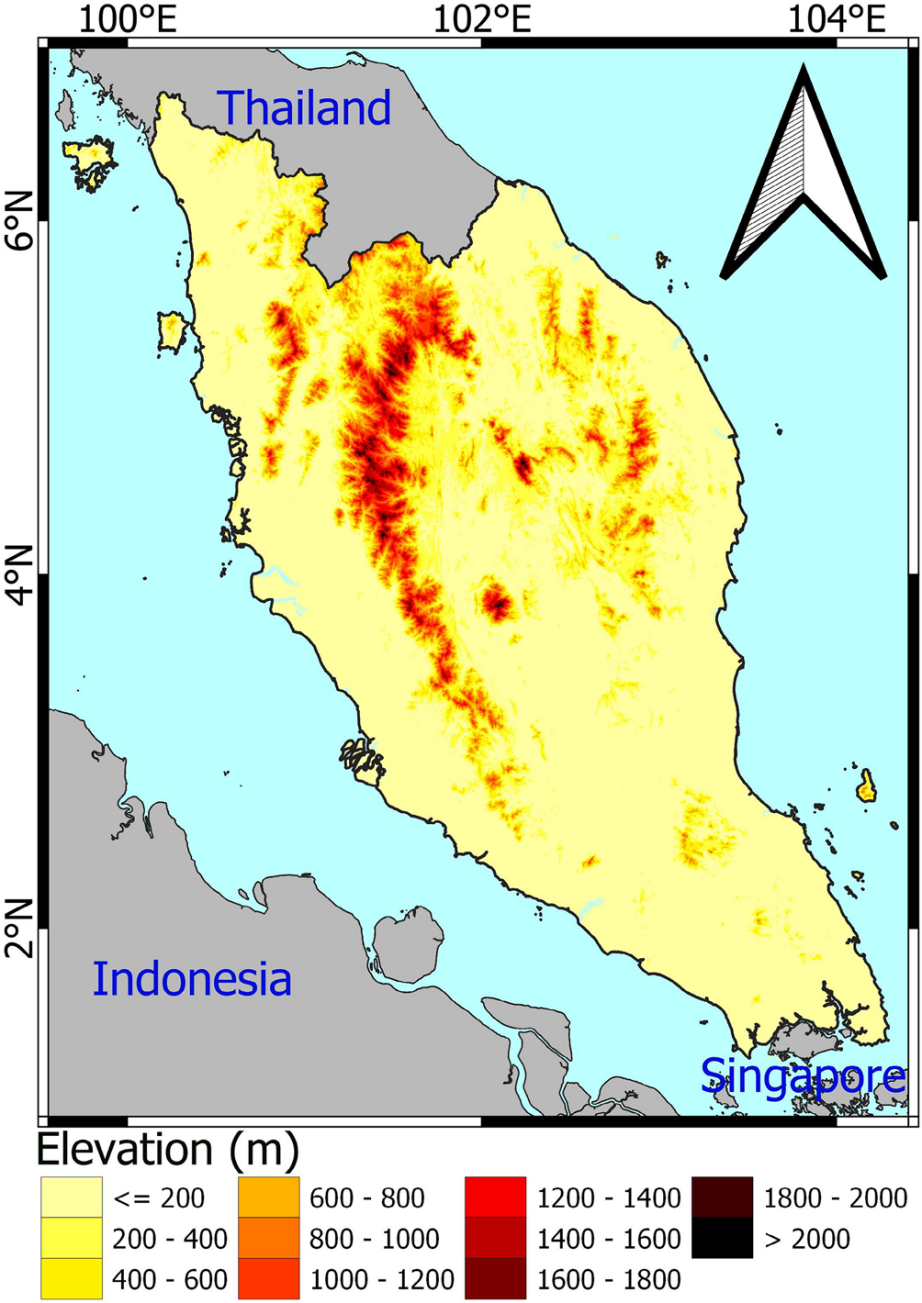
Elevation and geographical location of PM.

PM, bounded by latitudes 1.20‒6.40° N and longitudes 99.35‒104.20° E, has a tropical climate. It comprises a land of 130.6 thousand km^2^. PM is composed of uneven mountains covered with forests sloping towards seashores. PM's location in Southeast Asia is shown in Fig. 1. The climate of the PM is hot and humid. The temperature is more or less uniform throughout the year, ranging from 21.0 to 32.0 °C^44^. The yearly rainfall in PM ranges between 1800 and 4000 mm. Most of the area receives rainfall for more than 150 days a year.

**Figure 2:**
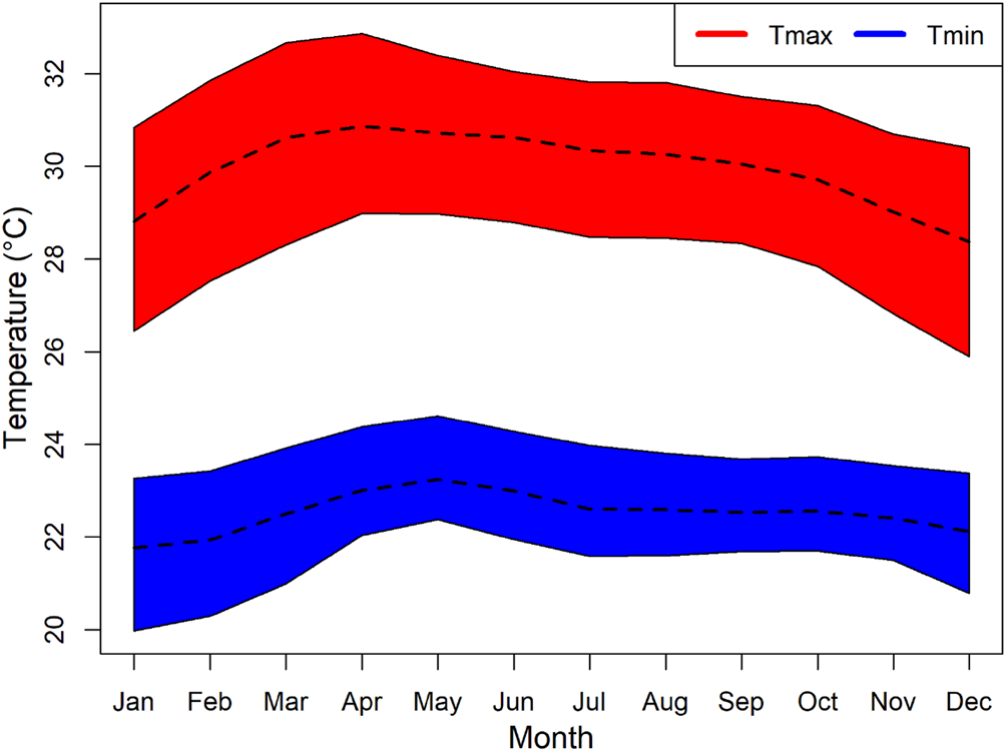
Variations in monthly Tmax and Tmin in PM for the period 1948 to 2016 based on ERA5 datasets with the 95th percentile confidence interval.

PM has two distinct monsoons blowing from southwest and northeast. The first lasts from May through August, while the second begins around November and continues for three to 4 months. PM experiences a dry climate during the first monsoon, particularly in the west and north. In contrast, the second one brings substantial rainfall, particularly in the country's eastern half. Looking at it on a broader scale, the Pacific Ocean is to the east of PM, while the Indian Ocean is to the west. As a result, natural climatic variability connected with these water bodies substantially impacts the PM climate^45^. Variations in maximum (Tmax) and minimum (Tmin) temperatures over a year are shown in Fig. 2. The daily Tmax in the study is the lowest in December (28.1 °C) and the highest in April (30.8 °C). The daily Tmin are also the lowest in January (21.8 °C) while highest in May (23.3 °C).

## Data and sources

The European Centre for Medium-Range Weather Forecasts (ECMWF) has released ERA5, a worldwide atmospheric reanalysis dataset. Weather balloons, satellites, and ground stations are all observational sources for this model. ERA5 provides a long-term (1950-present) global coverage of meteorological data with a horizontal resolution of 0.25° latitude–longitude, equivalent to roughly 27 km in peninsular Malaysia because of its proximity to the equator. The data is structured on a regular grid, where each data point is associated with a precise latitude and longitude coordinate. The ERA5 temperature data is accessible at various pressure levels as well as at the surface level, usually measured in °C. The ERA5 temperature data at a height of 2 m was obtained by downloading it from the Copernicus Climate Change Service website: https://cds.climate.copernicus.eu/cdsapp#!/dataset/reanalysis-era5-single-levels?tab=overview . The ERA5 data is accessible in several forms, such as NetCDF and GRIB. The project involved downloading ERA5 temperature data in NetCDF format and subsequently converting it to. CSV format using the ‘terra’ package in R for subsequent analysis (Supplementary information).

ERA5 dataset has been utilized extensively in recent climate research in Africa^46,47^, Asia^48–50^ and on the global scale^51,52^. Comparing four reanalysis climate datasets, Khadka, et al.^53^ found that ERA5 was superior in Asia. In their investigation of eight reanalysis datasets for Central Asia, Jiang, et al.^48^ concluded that ERA5 performed the best.

For this analysis, we use the LandScan Global population dataset from Oak Ridge National Laboratory (ORNL), made available by East View Information Services^54^ from 2000 to 2022. It offers annual population data with a geographical resolution of 30 arcseconds (1 km). Aggregating LandScan data to a resolution of 0.25 degrees using the R package "r.raster" ensured spatial consistency with ERA5. The population estimates were kept intact during the aggregation process by computing a weighted total from all grid cells that did not include NA. Follow this URL to obtain the dataset: https://landscan.ornl.gov/. As there is no specific database of heatwaves in Malaysia, the present study attempts to make a list of historical heatwaves in PM based on media reports. The list of reported heatwaves occurring dates, affected areas, and reported impacts are provided in Table 2. It shows no heatwave-related report before 2016. However, it has become apparent news in the last three consecutive years (2019 to 2021).

**Table 2:** Historical heatwaves in PM.

Date	Affected areas	Reported impacts
18 March 2016	Perlis, Kedah, Perak and Pahang (> 37 °C)	Heatwave Level 2^55^. No reported casualty
21 March 2016	North Malaysia (Kedah and Perlis) (> 37 °C)	Schools closed^56^. No reported casualty
23 March 2016	Segamat, Johor (35–37 °C)	One death^57^
28 March–28 April 2016	Pahang, Perlis, Pulau Pinang, Perak, Kelantan and Kedah (> 37 °C)	The health department got 200 hot weather casualty reports, covering 126 fatigues and 22 heatstroke incidences^58^
26 April 2016	Jitra, Kedah (35 °C)	One death^58^
25 February 2019	Kedah, Perak, Pahang, Kuala Lumpur and Johor (35–37 °C)	Most Peninsular Malaysia experienced dry conditions, with less rainfall and heatwave-like conditions. No reported casualty
17 March 2019	Perlis, Kedah, Perak, Pahang, Selangor, Negeri Sembilan, Malacca, Johor and Kuala Lumpur (35–37 °C)	Nine states (Perlis; Kedah; Perak; Pahang; Selangor; Negeri Sembilan; Malacca; Johor; KL) on level 1 and one state on level 2 (Pendang, Kedah). No reported casualty
25 January 2020	Kuala Muda, Kedah and Kuala Kangsar, Perak (35–37 °C)	Heatwave Level 1 declared. No reported casualty
02 March 2020	Northern peninsular Malaysia (35–37 °C)	Heatwave Level 1 declared. No reported casualty
06 March 2021	Kuala Lumpur, Petaling Jaya, Sepang, Tampin, Alor Gajah, Melaka Tengah, Yasin, Tangkak dan Batu Pahat (35–37 °C)	Heatwave Level 1 declared. No reported casualty

## Methodology

### Defining heatwave

A major problem in assessing heatwave is the absence of a well-accepted heatwave definition^31^. Several indices, developed based on the different heatwave characteristics, have been used to determine the severity of heatwaves. In this study, the heatwave was defined based on the thermal load. Different percentile thresholds of Tmax were used to estimate the spatial coverage of heatwaves. The heatwave was finally defined based on the threshold corresponding to the area that matches the areas historically considered prone to heatwave occurrence^17^. However, the temperature threshold for defining heatwave widely varies with the geography and climate of a region^17,59^. This emphasizes the necessity of exploring a wide range of thresholds to find the most appropriate one^60^. Therefore, this study considered three percentiles, 95th, 97.5th and 99th of Tmax. Following Khan, et al.^17^ and others, the heatwave was defined by examining the area affected by Tmax above three percentiles, 95th, 97.5th, and 99th, for three or more days in a row. Subsequently, the characteristics of heatwaves, including affected areas, duration, etc., were estimated for all years to assess their trends. This study considered 1961–1990 as the reference period to assess the changes in heatwave characteristics, as WMO^61^ suggested this period to estimate climate normal.

### Heatwave characteristics

Nine heatwave-related indices were employed to evaluate patterns in various heatwave features. The indices were selected based on the literature review^4,34,62,63^. Previous studies reported these nine indices to understand the severity of heatwave. The description of the indices is given in Table 3. The general conception of heatwave impacts on health consequences varies, but the vulnerable populations are usually most impacted when the temperature exceeds the thresholds during both day and night. Therefore, it is important to consider high temperatures both day and night to assess heatwave severity. Generally, temperature decline at night reduces its water vapour holding capacity and increases dew point temperature. The higher dew point can harm public health even with a lower temperature. Studies showed that most deaths during the European heatwave in 2003 were at night^38,64^. This emphasizes the assessment of heatwave indices considering both day and night temperatures. Several studies employed such indices for assessing changes in heatwave characteristics^65–68^.

**Table 3:** Definition of heatwave indices used in the present study.

Index	Description	Unit
AHW	Area experiencing Heatwave	km^2^
DHW	The maximum length of heatwaves	days
THW	Tmax in heatwave days	°C
CTHW	The growing temperature during heatwaves	°C
HWI	Heatwave index (% of the area covered by the number of heatwaves)	–
AHWN	The area experiences Tmax and Tmin more than a threshold	% of the total area
DHWN	Duration when Tmax and Tmin are more than a threshold	Days
THWN	Tmax during heatwaves at nighttime	°C
CTHWN	The cumulative temperature during heatwaves at nighttime	°C

### Trends in heatwave indices

Sen's slope estimator^69^ and the modified Mann–Kendall (MMK) trend test^70^ were used to assess the change rate and significance of heatwave indices, respectively. Sens slope estimates the rate of change of a series with *n* data points using the following equation,

1 $$Se{n}^\text{'}s\, Slope=\left\{\begin{array}{ll}{Q}_{(n+1)/2},& when\, n\, is\, odd\\ \frac{{Q}_{n/2}+{Q}_{(n+2)/2}}{2},& when\, n \,is\, even\end{array}\right.$$

Here, *Q* denotes the slope between two successive data points.

Serial correlation in climatic time series arises when consecutive data points exhibit dependence. This can artificially increase the MK statistic and result in spurious positive trends. The MMK test addresses this concern by including the autocorrelation coefficient (ρ) to modify the variance of the MK statistic. Thus, the MMK test offers a more reliable assessment of monotonic patterns in a series^18,71,72^. MMK test first de-trends the series if it detects a trend using the classical MK test and then estimates normal variant equivalents of the de-trended series. The Hurst coefficient (H) of the series with sample size *n* is determined with the subsequent formula:

2 $$H=log\left(\frac{S}{var\left(S\right)}\right)+0.5log\left(n\right)/log\left(n\right)$$

where S and var (S) is the MK statistic and its variance.

For a known H, the autocorrelation (pl) of lag l is calculated by solving the equation presented by Koutsoyiannis (2003).

3 $${\rho }_{l}=\frac{1}{2}\left({\left|l+1\right|}^{2H}-2{\left|l\right|}^{2H}+{\left|l-1\right|}^{2H}\right)$$

If H is found to be significant based on mean and standard deviation, the biased estimate of the variance of MK test statistic S for given H is calculated as,

4 $${Var(S)}^{{H}^{\prime}}={\sum }_{i<j}\bullet {\sum }_{k<l}\frac{2}{\pi }{sin}^{-1}\left(\frac{\rho \left|j-l\right|-\rho \left|i-l\right|-\rho \left|j-k\right|+\rho \left|i-k\right|}{\sqrt{(2-2\rho \left|i-j\right|)(2-2\rho \left|k-l\right|)}}\right)$$

where i, j, k and l are the indices representing different time points in the time series. The bias is then corrected by multiplying ${Var(S)}^{{H}^{\prime}}$ with correction factor and used to calculate the Z statistic to decide the significance of the trend based on Z statistics:

5 $${\text{Z}} = \left\{ {\begin{array}{*{20}l} {\frac{{{\text{S}} - 1}}{{\sqrt {{\text{Var}}({\text{S}})^{{\text{H}}} } }},} \hfill & {when \,S > 0} \hfill \\ 0 \hfill & {when\, S = 0} \hfill \\ {\frac{{{\text{S}} - 1}}{{\sqrt {{\text{Var}}({\text{S}})^{{\text{H}}} } }},} \hfill & {when \,S < 0} \hfill \\ \end{array} } \right.$$

A detailed methodology for trend analysis using the MMK test can be found in Ahmed et al. (2017) and Nashwan et al. (2019).

Sen's slope and the MMK test were computed using the "MannKendall" and "MannKendallLTP" functions from the "HKhkprocess" package in R. The *p*-value and Sen's slope are calculated at each grid point to implement the Mann–Kendall test under the scaling hypothesis. The alternative hypothesis is preferred when the probability value is smaller than the significance level (*p* < 0.05). The maps in the paper were prepared using the "Raster" package in R.

## Results

### Spatial distribution of daily maximum and minimum temperatures

**Figure 3:**
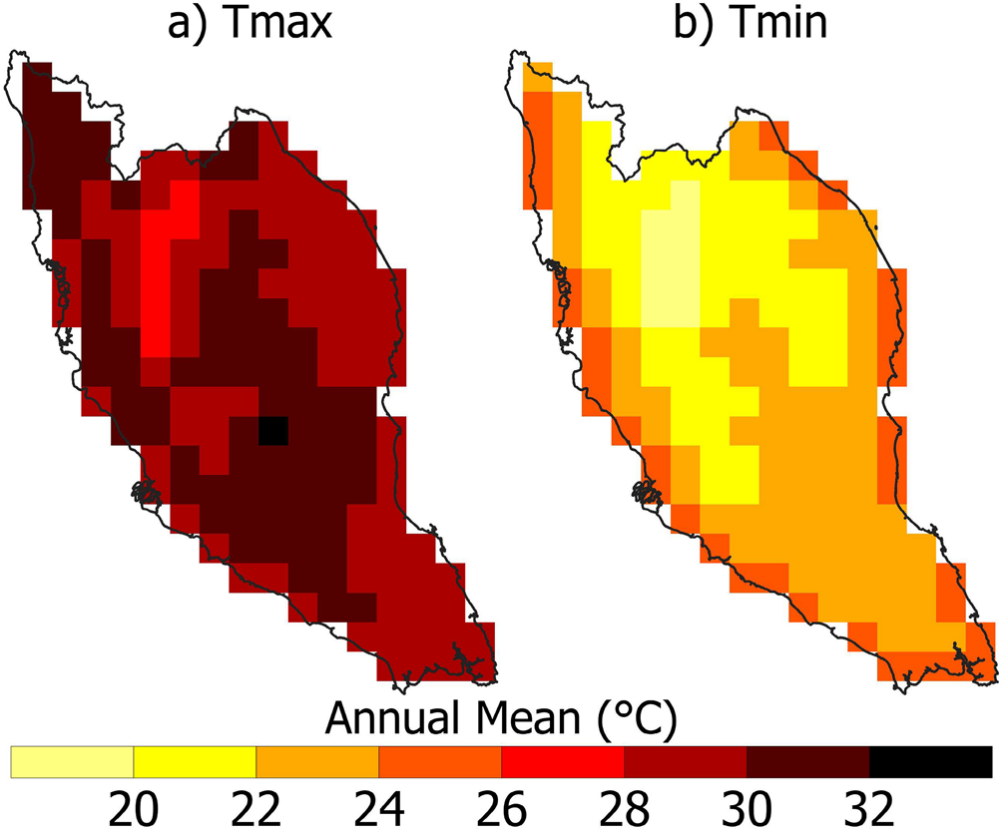
Spatial distribution of (**a**) Tmax (**b**) Tmin across PM based on ERA5 datasets for the period 1950 to 2022.

Figure 3 shows the geographical distribution of the yearly mean daily Tmax and Tmin in PM. The Tmax in the study area varies from 26 to 33 °C, while Tmin is between 16 and 26 °C. April, May and June are the hottest months of the year. PM experiences roughly six hours of direct sunshine daily, with cloud cover most probable during the afternoon or evening^73,74^. The higher temperature is generally on the coasts and the lower in the central highlands. The Tmax and Tmin datasets were used to estimate the heatwave indices mentioned in Table 3. All the indices were estimated based on the ERA5 dataset to calculate the affected area of heatwaves.

### Heatwave-affected areas

This study used three thresholds (95th, 97.5th and 99th percentile) across all grid points in each year to identify where heatwaves occurred based on those thresholds. This allowed the identification of the regions in PM that experienced heatwaves each year. Subsequently, each year's heatwave characteristics were estimated, generating the time series of characteristics like affected area, duration, and others. Trend analysis was then conducted on each characteristic to assess their changes over time.

**Figure 4:**
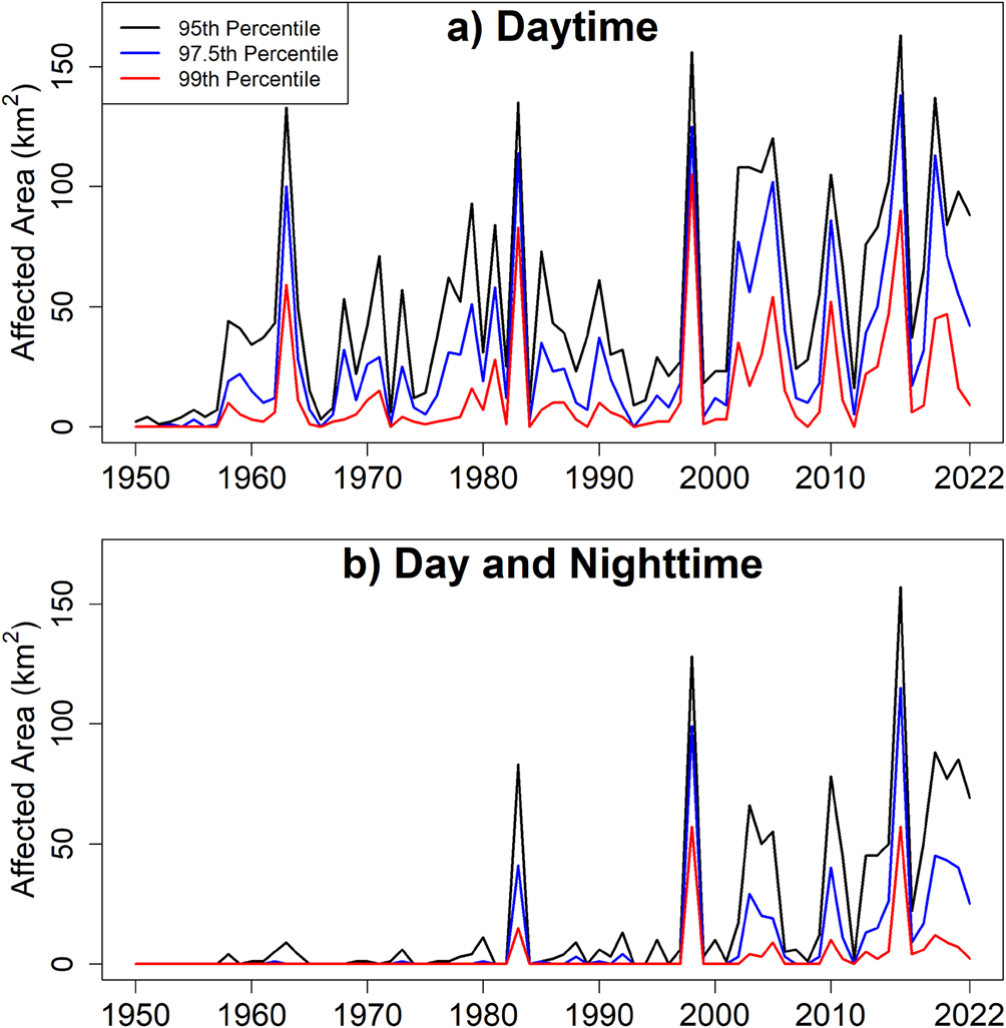
The affected area of heatwaves defined based on consecutive 3 or more days for (**a**) Tmax more than 95, 97.5 and 99-th percentiles and (**b**) Tmax more than 95, 97.5 and 99-th percentile and Tmin more than 95, 97.5 and 99-th percentile.

Figure 4a shows the heatwave-affected area based on successive three or more days with Tmax more than the 95th, 97.5th and 99th percentile temperature for the reference period (1961–1990). The affected area is presented on the Y-axis. A similar graph of heatwave-affected area based on exceedance of day and nighttime temperatures (Tmax and Tmin) higher than the 95th, 97.5th and 99th percentile temperature for successive three or more days is presented in Fig. 4b. Both figures show an annual temporal increase of heatwave-affected areas in PM. The pattern in the affected areas for the 97.5th and 99th percentile thresholds was similar to those obtained for the 95th percentile threshold. The heatwave-affected area increased for both percentiles. The results undoubtedly establish the increased heatwaves and the affected area in PM. Global warming has caused an increase in temperature across the world. This is also reported for PM^75–77^. The increase in temperature has caused an increase in heatwaves in PM. Most of the heatwave-affected area graphs in Fig. 4 show very little or no affected area before the 1980s (except 1963), while it became a common phenomenon in recent years (after 2000).

### Heatwave durations

**Figure 5:**
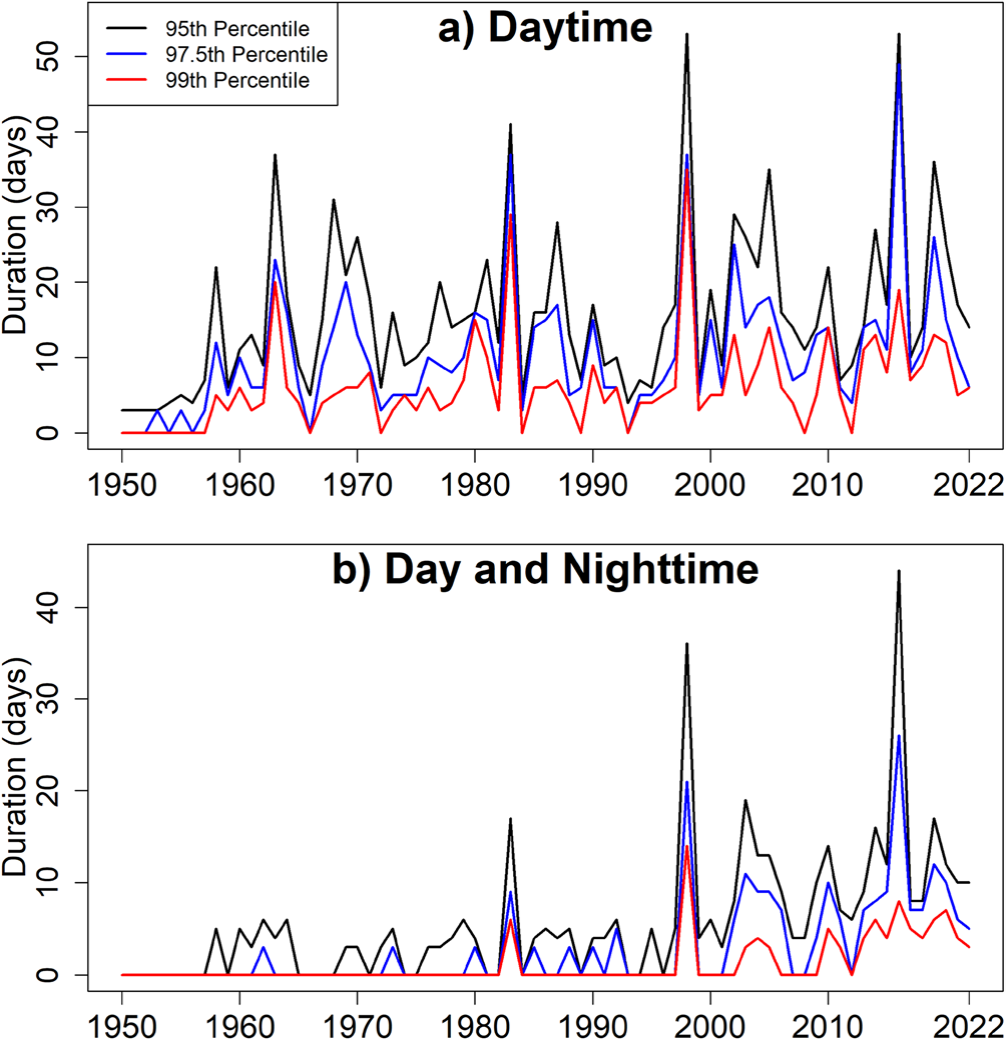
Duration of heatwaves defined for (**a**) Tmax more than 95, 97.5 and 99-th percentiles and (**b**) Tmax more than 95, 97.5 and 99-th percentile and Tmin more than 95, 97.5 and 99-th percentile.

Figure 5 shows the duration of heatwaves for different thresholds (95, 97.5 and 99th percentile) during daytime (upper panel) and both daytime and nighttime (bottom panel). The figures show a sharp increase in heatwave duration in PM. The increases were much more evident for higher thresholds of temperature. Figure 5b shows that exceeding more than the 99th percentile threshold for three or more consecutive days and nights never happened before 1982. However, such phenomena have occurred a few times in the last two decades. In this study, heatwave was also estimated based on the high day and night time temperatures. The results showed the increased frequency of such heatwaves with a duration of more than or equal to 3 days in recent decades. It means more PM populations have been vulnerable to heatwaves in recent years.

### Maximum temperature during heatwaves

**Figure 6:**
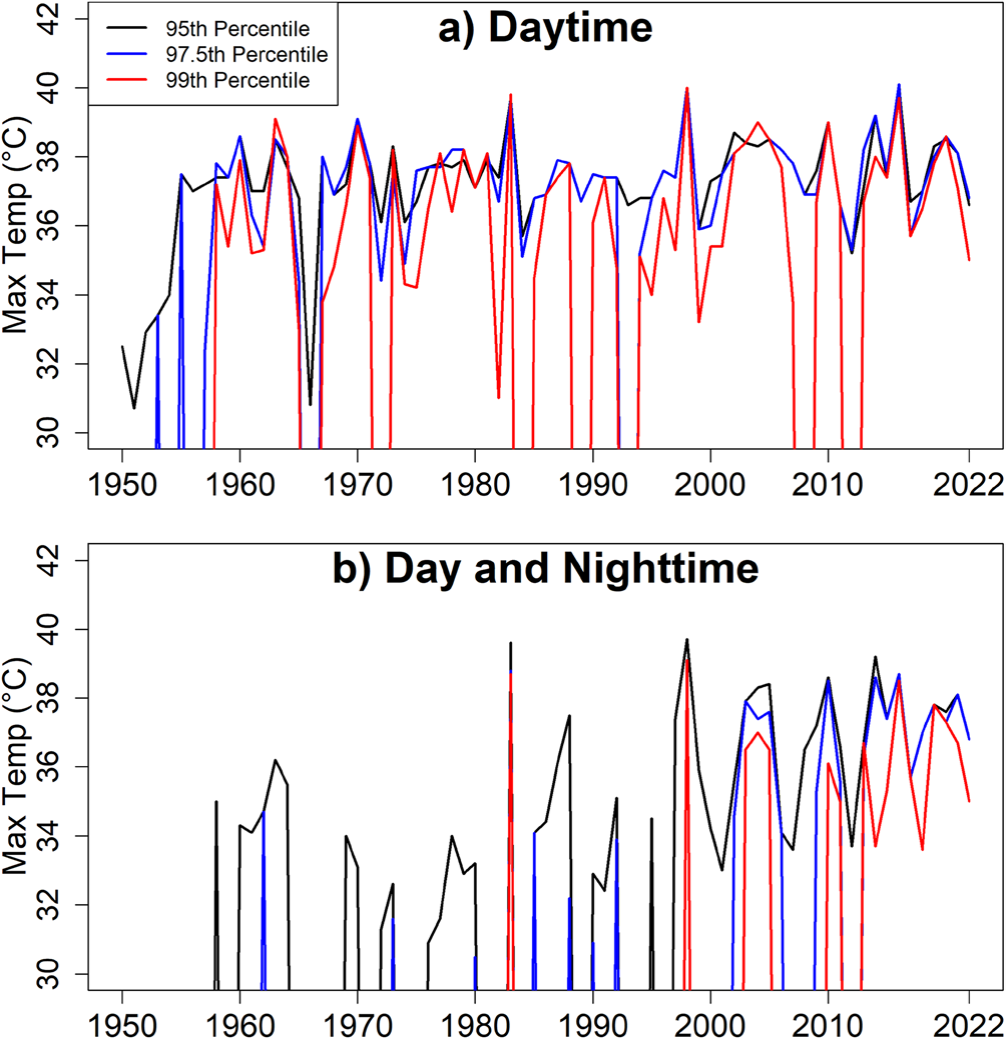
Tmax during heatwaves defined for (**a**) Tmax more than 95, 97.5 and 99-th percentiles and (**b**) Tmax more than 95, 97.5 and 99-th percentile and Tmin more than 95, 97.5 and 99-th percentile.

Figure 6 shows the maximum temperature during heatwaves for different thresholds (95, 97.5 and 99th percentile) during daytime and nighttime. The figure shows that heatwaves are increasing in terms of affected area, duration and severity. Tmax during heatwave days was much higher in recent years than in past years. This is particularly evident from the day and nighttime temperature graphs in Fig. 6b. The event was found to be correlated with weak (2004–05), moderate (2009–10), and very strong (1997–98) El Niño events^78,79^. Recent fast temperature rise may cause such an increase in temperature during heatwave periods. Earth's temperature is projected to increase unceasingly due to global warming. This will certainly make the heatwaves more intense in the future.

### Heatwave index

**Figure 7:**
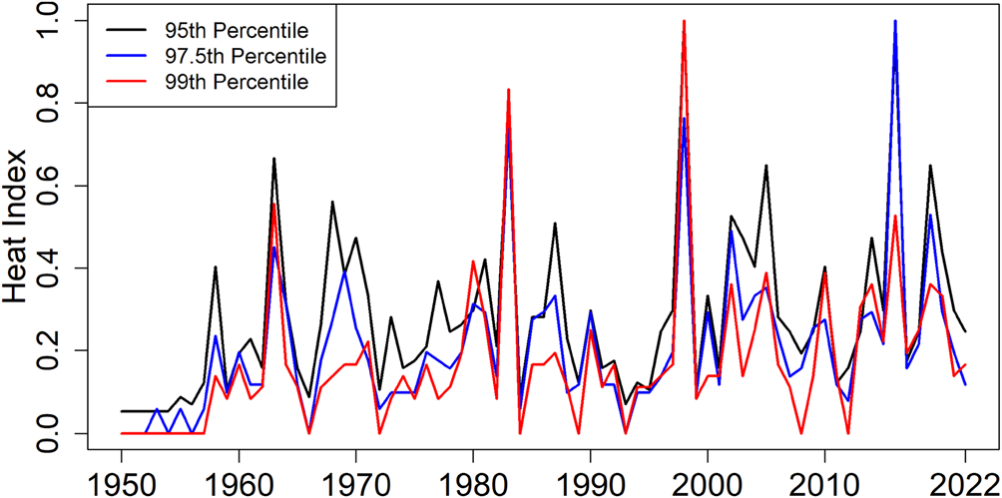
Time series of heatwave index for Tmax > 95th percentile (black line); Tmax > 97.5th percentile (blue line); Tmax > 99th percentile (red line).

The heat index (HI) provides an integrated measure of heatwave-affected area, duration and intensity. It is estimated as the fraction of the total area of PM multiplied by the number of heat waves. Therefore, this study also estimated and presented the heat wave index for different thresholds (95, 97.5 and 99th percentile). The obtained results in Fig. 7 show a rise in the heat index. The highest value (HI > 0.8) for the 95th percentile resulted in 1983, 1998 and 2016. The HI > 0.8 occurred only in 1983 for both 95th and 97.5th percentiles, while in 1983 and 1998 for the 99th percentile.

The trends in different heatwave characteristics are presented in Table 4. The table shows the trends for the heatwave defined based on 95, 97.5 and 99th percentiles. The table shows that heatwaves affected area, heat duration, maximum temperature during heatwaves, cumulative temperature during heatwaves, and HI, which increased significantly in the study area. The heatwave-affected area in PM has increased significantly by 8.98 km^2^/decade and duration by 1.54 days/decade when it is defined based on the 95th percentile of Tmax as a threshold. Also, the night cumulative temperature during the heatwave was increased by 46.7 °C/decade.

**Table 4:** Trends (changes/decade) in different heatwave characteristics in PM.

Heatwave characteristics	95th percentile		97.5th percentile		99th percentile	
	Change	*p*-Value	Change	*p*-Value	Change	*p*-Value
Affected area (km^2^)	**8.98**	0.0	**5.2**	0.0	**1.71**	0.0
Duration (day)	**1.54**	0.003	**1.22**	0.0	**0.95**	0.0
Maximum Temp (°C)	**0.17**	0.05	0.28	0.45	**0.5**	0.05
Cumulative Temp (°C)	2.45	0.89	1.4	0.98	0.0	0.93
Heat Index	**0.03**	0.004	**0.02**	0.0	**0.026**	0.0
Affected Area (Night) (km^2^)	**2.98**	0.0	**0.27**	0.0	0.0	0.0
Duration (Night) (day)	**1.4**	0.0	0.0	0.0	0.0	0.0
Maximum Temp During HW (Night) (°C)	**1.47**	0.0	0.0	0.0	0.0	0.0
Cumulative Temp During HW (Night) (°C)	**46.7**	0.0	0.0	0.0	0.0	0.0

The bold number represents a significant change at *p* < 0.05.

### Spatial distribution of affected area

**Figure 8:**
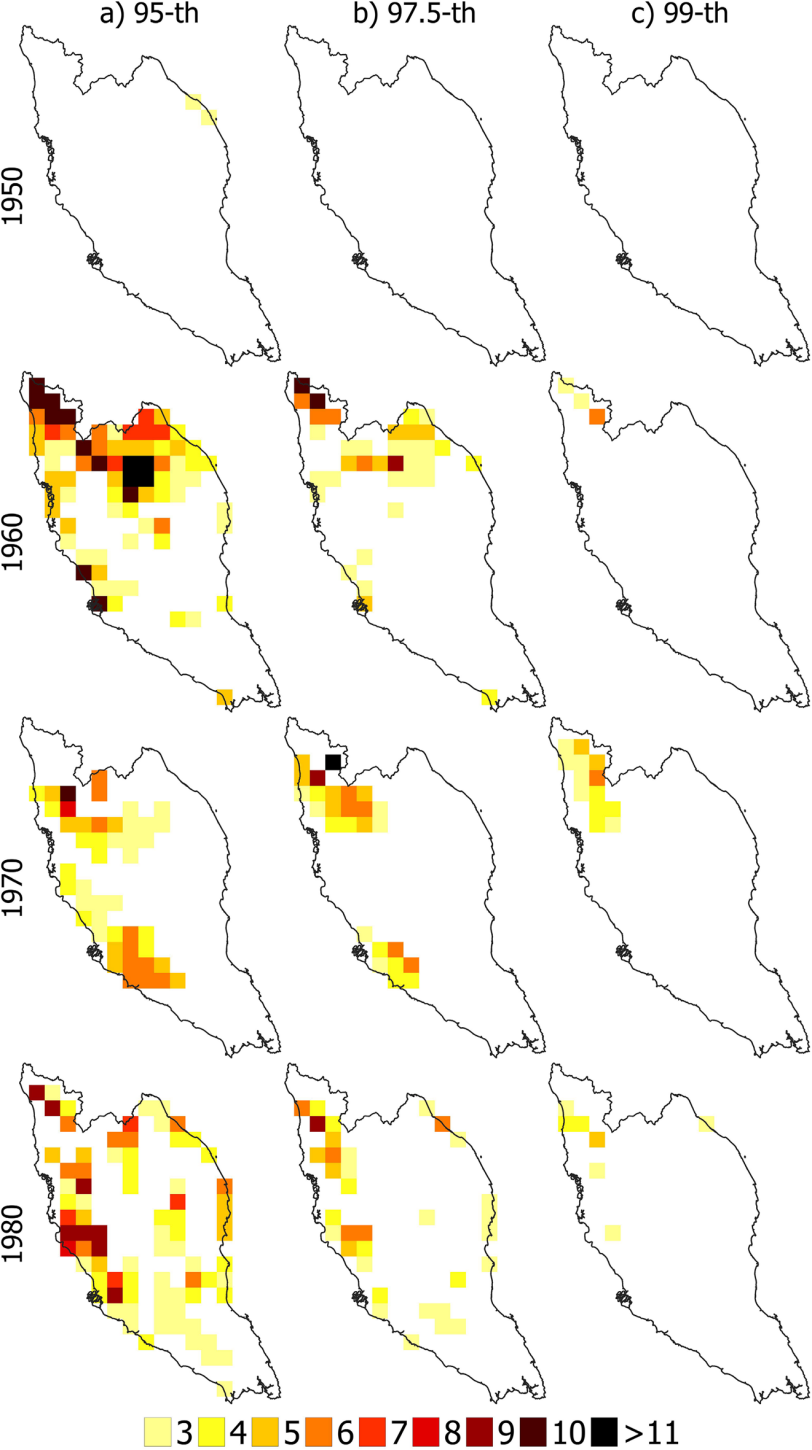
Spatial distribution of heatwaves when defined based on 95th percentile (left panel), 97.5th percentile (middle panel) and 99th percentile (right panel) threshold for the decades 1950, 1960, 1970 and 1980.

**Figure 9:**
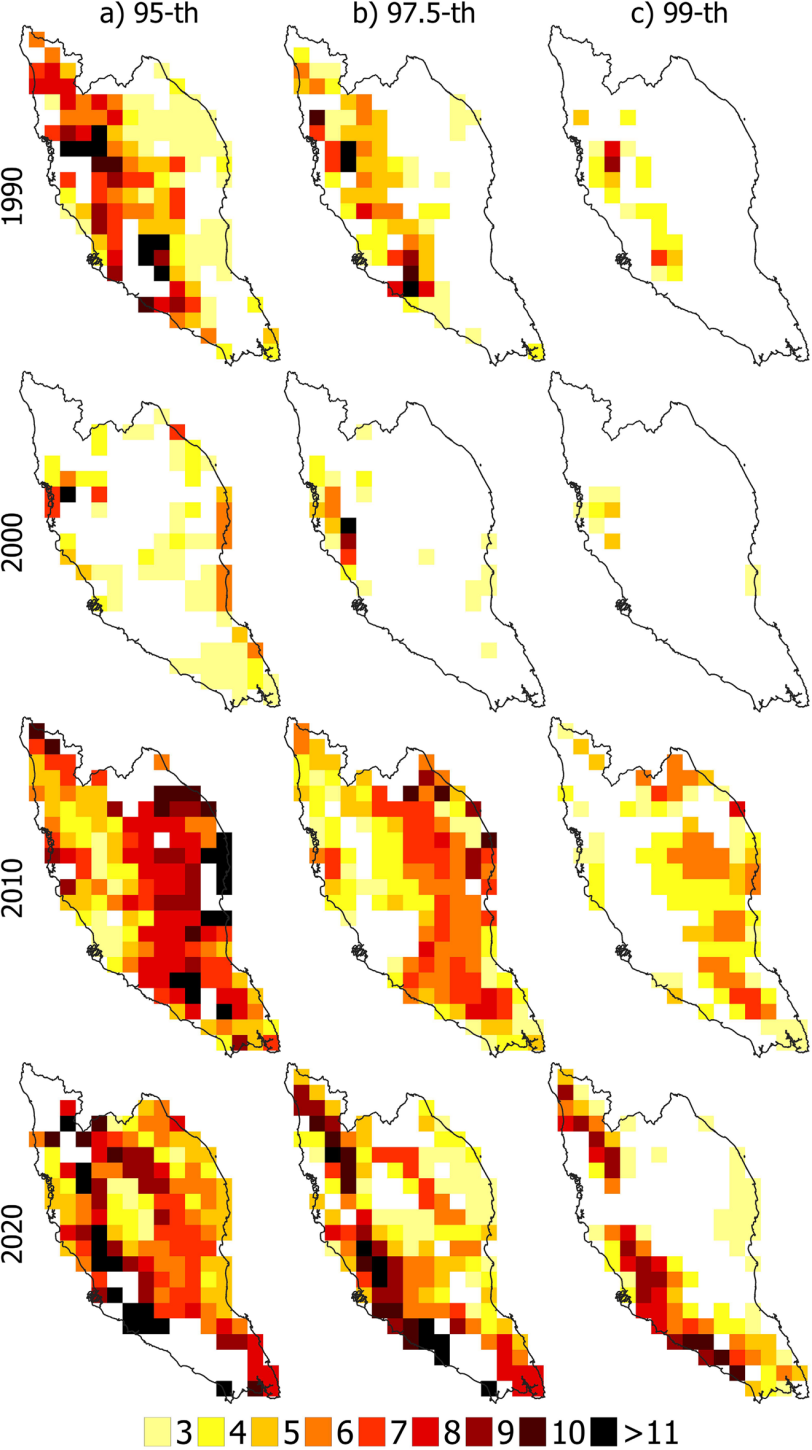
Spatial distribution of heatwaves when defined based on the 95th percentile (left panel), 97.5th percentile (middle panel) and 99th percentile (right panel) threshold for the decades 1990, 2000, 2010 and 2020.

Figures 8 and 9 show the spatial distribution of heatwave-affected regions over PM for decades 1950, 1960, 1970, 1980, 1990, 2000, 2010 and 2020 using 95, 97.5, and 99th percentile thresholds. The results revealed that heatwave-affected covered almost the whole PM for all years between 1990 and 2020 if the threshold was 95th percentile. Nearly half of the area was affected in 1990 when heatwave was defined based on the 97.5th percentile threshold. The heat wave impacted only the eastern region of PM in 2000, while certain eastern and southern areas were unaffected in 2010 and 2020. Similarly, not all affected heatwave areas were covered when the 99th percentile threshold was used. For example, in 1990 and 2000, most of the areas in PM did not experience a heatwave, while in 2010, the eastern part experienced a heatwave. It should be noted that high temperatures due to the moderate El Niño event contributed to heatwave in 2010. Therefore, the 99th percentile threshold was unsuitable for defining heatwave and the affected area, while the 97.5th percentile was more suitable. However, when the 95th percentile of Tmax was the threshold, the geographical coverage of the heatwaves matched reasonably (almost all the region) with the historical heatwave-affected PM area. This indicates that three consecutive days with Tmax above 97.5th percentile can best define a heatwave in PM.

### Population exposure to heatwaves

**Figure 10:**
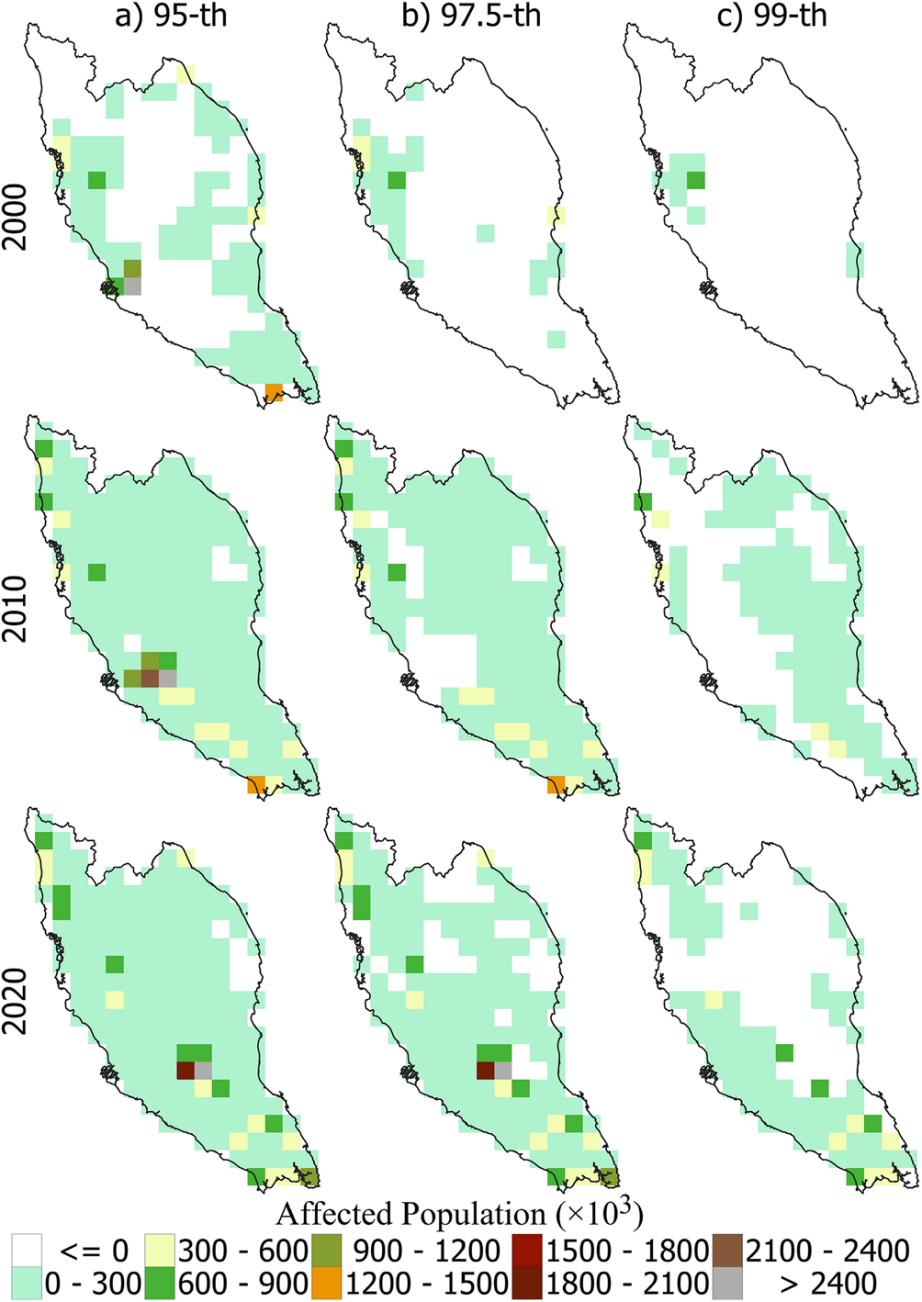
Spatial distribution of affected population to heatwaves when defined based on 95th percentile (left panel), 97.5th percentile (middle panel) and 99th percentile (right panel) threshold for the years 2000, 2010 and 2020.

Figure 10 shows the spatial distribution of the population in peninsular Malaysia affected by heatwaves in 2000, 2010, and 2020, defined using the 95th, 97.5th, and 99th percentiles of Tmax. The figure reveals a gradual increase in population exposure to heatwaves. For the 95th percentile, only the coastal region was affected in 2000. However, by 2010, the entire peninsula was affected. A similar increase was observed for the other two thresholds. In all cases, the highest population exposure was in the south, with some grids affected by more than 2,400,000 people. In contrast, the northeast was the least affected region.

**Figure 11:**
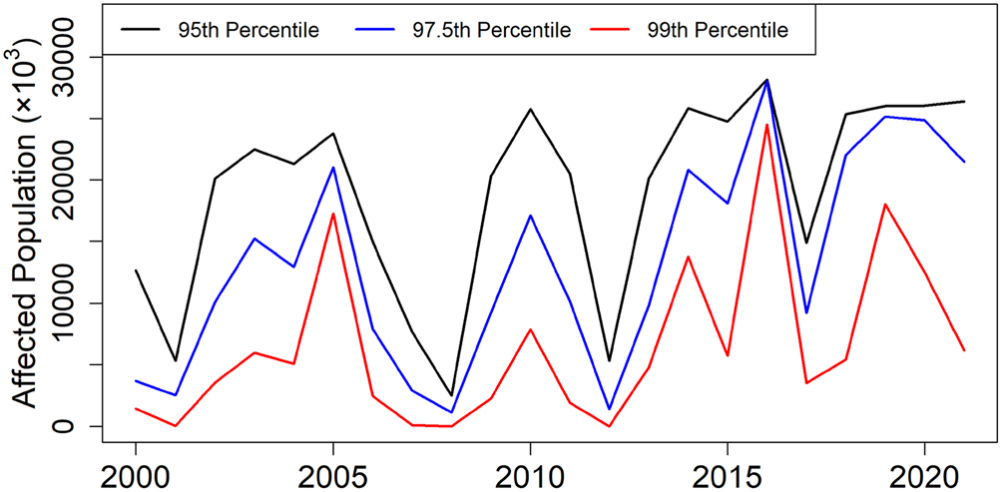
Time series of the affected population to heatwaves for Tmax > 95th percentile (black line); Tmax > 97.5th percentile (blue line); Tmax > 99th percentile (red line) from 2000 to 2022.

Figure 11 shows the time series of the population affected by heatwaves from 2000 to 2022, defined using the 95th, 97.5th, and 99th percentiles of Tmax. The figure shows significant fluctuations in the affected population over the study period due to the natural variability of Tmax in peninsular Malaysia. The years with the highest affected population were 2005, 2010, 2016, 2018, 2019, 2020, and 2021, with 2016 having the highest affected population. However, all three lines in the figure show a gradual increase in the affected population over time, with a higher increase observed for heatwaves defined using the 97.5th and 99th percentiles of Tmax.

The findings of this study suggest that heatwave exposure is increasing in peninsular Malaysia, both spatially and temporally. This is likely due to various factors, including climate change, population growth, and urbanization. The population exposure map generated in this study is important to take steps to mitigate the risks of heatwaves and protect vulnerable populations.

## Discussion

This study used the daily ERA5 temperature dataset with a geographical resolution of 0.25° for 1950–2022 to analyze the changes in the frequency and severity of heat waves across PM. Houmsi, et al.^73^ assessed the performance of ERA5 in PM compared to station data. The results demonstrated ERA5's strong capacity to replicate a variety of variables across all stations, indicating the reliable performance of ERA5 in PM. Besides, et al.^80^ evaluated ERA5 over the Kelantan River Basin (North Malaysia) and concluded its ability to capture the climatological patterns of temperature variables reasonably. The time series of ERA5 global surface temperature anomalies show that temperatures were relatively stable from 1950 until the late 1970s^81^. There are significant differences in the accuracy in different regions, with Europe being well represented in the early period but Australia being less. However, the ERA5 monthly precipitation variability agrees with observations in all continents, with correlations above 90% for most of Europe and typically above 70% for North America, Asia, and Australia.

Geographical and climatic factors greatly affect the characteristics of heatwaves. This has made it challenging to define a region's heatwave. Heatwave has been defined in the literature using a variety of techniques. The thermal load approach, however, has been the most widely used due to its benefits, including the ability to assess changes in heatwave characteristics or compare heatwaves among different regions, which are crucial for planning adaptation strategies and providing relief during a heatwave. Regardless of the techniques employed to define heatwave, confirming the obtained definition is the main difficulty. This study relied on reports of heatwave incidence and media coverage to validate the definition. In literature, several researchers have employed the historical, cultural, or general perception of the region afflicted by heatwave in defining it^17,59^. Nairn and Fawcett^59^ used historical knowledge of heatwave-affected areas to define heatwaves in Australia. et al.^17^ validated the concept of a heatwave in Pakistan using heatwave reports from newspapers and official reports. In countries without accurate historical records of droughts, a similar idea has also been employed to define droughts. For instance, Shahid^82^ used the area identified as being affected by the drought in the newspaper to validate the drought map of Bangladesh. Ahmed, et al.^83^ also used reports in the public media to validate the boundaries of the drought-prone zones determined by drought indices. The study showed that the definition derived in this study demarcated the heatwave-affected areas that matched the reported heatwave-affected area.

Despite growing concern about heat stress, studies on heatwaves are very few in Malaysia^22,84^. Suparta and Yatim^22^ attempted to characterize heatwave in PM. However, they used the excess heat factor to define heatwave, which is unreliable as the heat factor varies from location to location. They also used a few in-situ data that could not estimate heatwave-affected areas or their geographical variability. However, they also reported 2005 and 2010 as the hot years during the short period of data they analyzed. The geographical distribution of historical heatwave-affected areas reconstructed in this study indicates that the north of PM suffered more frequent and longer-lasting heatwaves than other parts. This agrees with Kamal, et al.^84^. They used the extreme heat index to show the most extreme heat-affected PM areas and showed that the northern PM region is most vulnerable.

The present study showed a rise in different heatwave characteristics in PM. Time series analysis of heatwaves in nearby regions also revealed a similar result. Pimonsree, et al.^85^ revealed more recurrent heatwaves in Thailand. One of the major findings of this study is the increasing trends in heatwave frequency, duration and cumulative temperature during affected days. This agrees with the studies conducted in Southeast Asia^86,87^. Dong, et al.^87^ evaluated the trends in heatwave characteristics over Southeast Asia and showed more recurrent and longer-duration heatwaves in the region. They also showed higher temperatures during the affected period. Li^86^ also reported a higher incidence and longer-period heatwaves in Southeast Asia in recent years than before.

This study showed a gradual increase in population exposure to heatwaves in peninsular Malaysia, both spatially and temporally. This implies the emergence of a pressing issue that necessitates attention and resolution. These findings are consistent with previous research in Southeast Asia^87–89^. The potential adverse effects on public health, particularly among vulnerable populations such as the elderly, individuals with low socioeconomic status, and those with pre-existing health issues, are expected to arise from the escalating incidence of heatwaves and subsequent population exposure. Heatwaves can exert a substantial economic influence by causing disruptions to enterprises and diminishing output.

The climate model's simulations indicated continuous global temperature rise, making the heatwaves more recurrent and intense. The projection of heatwaves under rising temperatures is not the scope of this study. However, the reviews of such studies in the nearby countries revealed a gradual increase in heatwaves recurrence and duration in the region, like in other parts of the globe^87,90^. An earth system model simulation conducted by Dong, et al.^87^ revealed a high incidence of longer-lasting and higher intense heatwaves in Southeast Asia in the future. Rising heatwaves could considerably affect public health and well-being in the future. Arsad, et al.^91^ conducted a literature review to report the implications of heatwaves and showed a significant increase in mortality during heatwaves. Due to its tropical location, Malaysia may experience catastrophic heatwaves in the future. The maps produced by this work can help with regional planning to lessen the consequences of heatwaves.

## Conclusions

This study assessed and mapped the temporal and geographical variability of heatwaves in PM. The main outcome is deriving a suitable definition of a heatwave for PM. The spatial and temporal variability analysis of heatwaves, defined based on three thresholds, indicates that three consecutive days with Tmax above 97.5th percentile can best determine a heatwave in PM. The spatial distribution of historical heatwave-affected areas approximately matched the areas mapped using the derived definition. Therefore, it can be used for monitoring heatwaves in PM. The present study also showed an increase in different properties of heatwaves, including affected area, duration, and severity. Overall, the heatwave affected area, and its duration increased rapidly. The geographical distribution of the reconstructed historical heatwaves revealed that the northern peninsula experiences more heatwaves. However, the southern region is also affected by recent heatwaves, indicating the gradual spread of the affected area over the entire peninsula. The spreading became more pronounced after 2000. Assessment of population exposure to heatwaves revealed a gradual increase both spatially and temporally. The spatial distribution of the population exposure identified in this study can help to take steps to mitigate the risks of heatwaves. Increasing heatwaves in PM may be linked to rising temperatures due to climate change. PM temperature is increasing with global temperature, which is supposed to continue into the present century. Therefore, PM may experience more frequent heatwaves in the coming years. The country must take the required adaptation actions to lessen the impacts of climate change. However, the present study revealed no changes in heatwave indices considering both day and night temperatures. This indicates no significant changes in extreme night temperature in PM. In the future, other temperature products can be used to estimate uncertainty in the heatwave trends and their spatial distribution. Higher resolution data can be employed up to the present year to know the most recent changes in heatwaves in PM. The thresholds can be refined based on additional parameters like humidity to optimize heatwave detection further. The role of local factors like land-use change, urbanization, and deforestation can be investigated in exacerbating heatwaves. Besides, the influence of El Nino and La Nina episodes on heat wave fluctuation in PM can be investigated. Statistical models can be developed to quantify the relative contributions of climate change and El Nino/La Nina to heatwave trends. The projections of global climate models can be downscaled to provide high-resolution projections of future heatwave patterns in PM. Increased population is also a cause of increased population exposure to heatwaves. Future studies can quantify the contributions of population growth and rising temperature separately to accurately understand climate change's impacts and other factors.

### Supplementary Information

Supplementary Information 1.

Supplementary Information 2.

Supplementary Information 3.

Supplementary Information 4.

## Data Availability

The datasets used in the current study are available from the corresponding author upon reasonable request.
